# Supplementary material for: Multimodal electrophysiological analyses reveal that reduced synaptic excitatory neurotransmission underlies seizures in a model of NMDAR antibody-mediated encephalitis
Source: Commun Biol. 2021 Sep 20;4:1106. doi: 10.1038/s42003-021-02635-8 (PMC8452639; doi:10.1038/s42003-021-02635-8)
Supplement: Supplementary file 2 — Description of Supplementary Files [file 42003_2021_2635_MOESM2_ESM.pdf]

Description of Additional Supplementary Files

File name: Supplementary Movie 1

Description: Hyperexcitable phenotype captured 48 hours after NMDAR antibody ICV injection in juvenile Wistar rats.

File name: Supplementary Movie 2

Description: Example video clips of epileptic myoclonic jerks in NMDAR antibody chronically infused juvenile Wistar rats during wakefulness

File name: Supplementary Movie 3

Description: Example video clips of hyper excitability and epileptic myoclonic jerks in NMDAR antibody chronically infused juvenile Wistar rats during wakefulness.

File name: Supplementary Movie 4

Description: Example video clips of epileptic activity during sleep in NMDAR antibody chronically infused juvenile Wistar rats.

File name: Supplementary Data 1
Description: Source data underlying graphs and charts.
