# Supplementary material for: Multimodal electrophysiological analyses reveal that reduced synaptic excitatory neurotransmission underlies seizures in a model of NMDAR antibody-mediated encephalitis
Source: Commun Biol. 2021 Sep 20;4:1106. doi: 10.1038/s42003-021-02635-8 (PMC8452639; doi:10.1038/s42003-021-02635-8)
Supplement: Supplementary file 3 — Supplementary Information [file 42003_2021_2635_MOESM3_ESM.pdf]

Supplementary Information for:

Multimodal electrophysiological analyses reveal that reduced synaptic excitatory neurotransmission underlies seizures in a model of NMDAR antibody-mediated encephalitis

*Sukhvir K Wright,[1,2] †Richard E Rosch,[3,4,5] Max A Wilson,[1] Manoj A Upadhya,[1] Divya R Dhangar,[1] Charlie Clarke-Bland,[1] Tamara T Wahid,[1] Sumanta Barman,[6] Norbert Goebels,[6] Jakob Kreye,[7,8,9] Harald Prüss,[7,8] Leslie Jacobson,[10] Danielle S Bassett,[5,11] Angela Vincent,[10] Stuart D Greenhill,[1] *Gavin L Woodhall.[1]

1. Institute of Health and Neurodevelopment, College of Health and Life Sciences, Aston University, Birmingham, UK
2. Dept. of Paediatric Neurology, The Birmingham Women's and Children's Hospital NHS Foundation Trust, Birmingham, UK
3. MRC Centre for Neurodevelopmental Disorders, King's College London, London, UK
4. Department of Paediatric Neurology, Great Ormond Street Hospital for Children NHS Foundation Trust, London, UK
5. Department of Bioengineering, University of Pennsylvania, Philadelphia PA, USA
6. Department of Neurology, Medical Faculty, Heinrich Heine University Düsseldorf, Düsseldorf, Germany
7. German Center for Neurodegenerative Diseases (DZNE) Berlin, Germany
8. Department of Neurology and Experimental Neurology, Charité – Universitätsmedizin Berlin, Germany
9. Department of Pediatric Neurology, Charité – Universitätsmedizin Berlin, Germany
10. Nuffield Department of Clinical Neurosciences, Oxford University, Oxford
11. Santa Fe Institute, Santa Fe NM, USA

Supplementary methods

Dynamic causal modeling

Feature extraction from whole-cell patch clamp recordings

We quantified amplitude, decay time, and variability of sEPSCs measured from the whole-cell hippocampal patch clamp recordings described above. We selected 10 minutes of continuous, artefact free recordings from both control antibody and NMDAR antibody conditions, and bandpass filtered these epochs between 0.3Hz and 2000Hz. In these traces, we identified peaks with a local prominence of at least 20mV (n=1624 in control antibody condition; n = 374 in NMDAR antibody condition), and segmented windows of 30ms prior to the peak, and 60ms following the peak, of which we considered the first 20ms as baseline. We normalised each segment to the baseline mean and extracted the mean amplitude of these normalised peaks. For each segment we identified the time elapsed from the peak to when the signal had decayed to half-peak values, and we then calculated the mean of this period as the time constant of sEPSCs. To quantify variance in the responses, we calculated the cumulative frequencies of sEPSCs of given amplitudes (normalized to the maximal amplitude observed in each condition, respectively). We then quantified the gradient of this distribution at the 50th centile to approximate the population parameter σ used in DCM. Log-differences between these average quantitative features in the control antibody and NMDAR antibody conditions from the whole-cell patch clamp recordings were subsequently used to inform priors of DCM inversions of LFP data.

LFP segmentation and data extraction

We selected six 1 hour segments for 2 animals treated with control IgG, and three 1 hour segments for 1 treated with NMDAR antibody IgG, at the peak of the observed epileptogenesis (that is, at 48h). Each trace was z-scored and sections exceeding an absolute z-score of 5.5 were coded as seizure; else they were coded as interictal. We then added the 45s before and after the automatically identified seizure-segments to the seizure segments to capture seizure onset and seizure offset transitions. This process served to automate the LFP ictal/interictal

classification confirmed by visual analysis, and we then divided all segments into 45s sections for subsequent DCM analysis. In total this process resulted in 514 control antibody interictal segments, 0 control antibody seizure segments, 114 NMDAR antibody interictal segments, and 228 NMDAR antibody seizure segments after exclusion of artefact. For each of the LFP traces, average power spectra were estimated using a multivariate autoregressive model implemented in the DCM software[1].

DCM fit to control data

Assuming that data recorded from control antibody injected mice are representative of the 'baseline' state, we first fitted a single canonical microcircuit model (CMC)[2] using standard DCM inversion techniques (EM algorithm performing gradient descent on a free energy approximation of the negative log likelihood). This process provides us with posterior densities over neuronal parameters for the CMC (as summarized in Table 1).

DCM fit to NMDAR antibody interictal data

In order to test whether the synaptic changes identified in the whole-cell patch clamp recordings contribute to the LFP features recorded in NMDAR antibody injected mice, we fitted single CMC models under different prior parameter sets to the NMDAR antibody interictal LFP data and compared their relative evidence. The null model used control antibody derived parameter values exactly as priors, without any additional changes. The remainder of the model space was defined by altering prior parameter values quantitatively based on the expected control antibody *vs.* NMDAR antibody differences derived from the whole-cell patch clamp recordings. Specifically, we added the log difference between NMDAR antibody and control antibody sEPSC amplitude to excitatory coupling parameters $\gamma_{1\text{-}3}$; the log difference between NMDAR antibody and control antibody sEPSC half-life to the excitatory time constants $\tau_{2,3}$; and the log difference in cumulative variance between NMDAR antibody and control antibody size distribution to the population variance parameter σ. We divided the model space to compare models where only subsets of these changes were made on the priors. These were divided along two main design features: parameter type (γ, τ, σ, or their combinations) and location in the microcircuit (superficial, fast oscillator pair; deep, slow oscillator pair; or both), resulting in

a total of 7 x 3 models that carried some microscale information in their priors; and one null model without the microscale information. Comparison between these models was then made based on the free energy approximation of their respective model evidence[3]. The winning model was used for subsequent analyses.

<u>DCM fit to NMDAR antibody seizure data</u>

To identify parameter changes associated with the transition into epileptic seizures, we fitted a single CMC to NMDAR antibody seizure LFP segments using parameters from the winning model identified in the previous step. We considered the absolute parameter differences between the DCM fit to NMDAR antibody interictal data, and the DCM fit to NMDAR antibody seizure data as the parameter change associated with ictogenesis for the subsequent simulations.

Simulations

The parameters inferred by fitting the microcircuit models to LFP data are fully generative and can therefore be used to simulate novel data. We exploited this 'simulation mode' to test the effects of different gradual changes in parameters on the simulated LFP power spectrum. We ran simulations along two sets of parameter changes: 'epileptogenesis' parameters θ_E (i.e. the difference between control antibody and NMDAR antibody interictal estimated parameters); and 'ictogenesis' parameters θ_I (i.e. the difference between MMDAR antibody interictal and NMDAR antibody seizure estimated parameters). Simulations were in *n* = 200 steps along each of those axis, so that $\theta_{Sim}^{i,j} = \theta_{base} + \frac{i}{n}\theta_E + \frac{j}{n}\theta_I$ with θ_{base} representing the empirical parameters inferred from the control antibody model inversion (Equation 1).

We classified each simulated power spectrum across this parameter space based on the least squares difference to any of the three empirically observed states into control antibody-like, NMDAR antibody interictal-like, and NMDAR antibody seizure-like states. To quantify local changes induced by small changes in ictogenicity, we calculated the mean squared difference between (i) the power spectra derived at a given simulation parameterization, and (ii) the power spectra derived at neighbouring parameterizations along the θ_I direction.

Supplementary figures

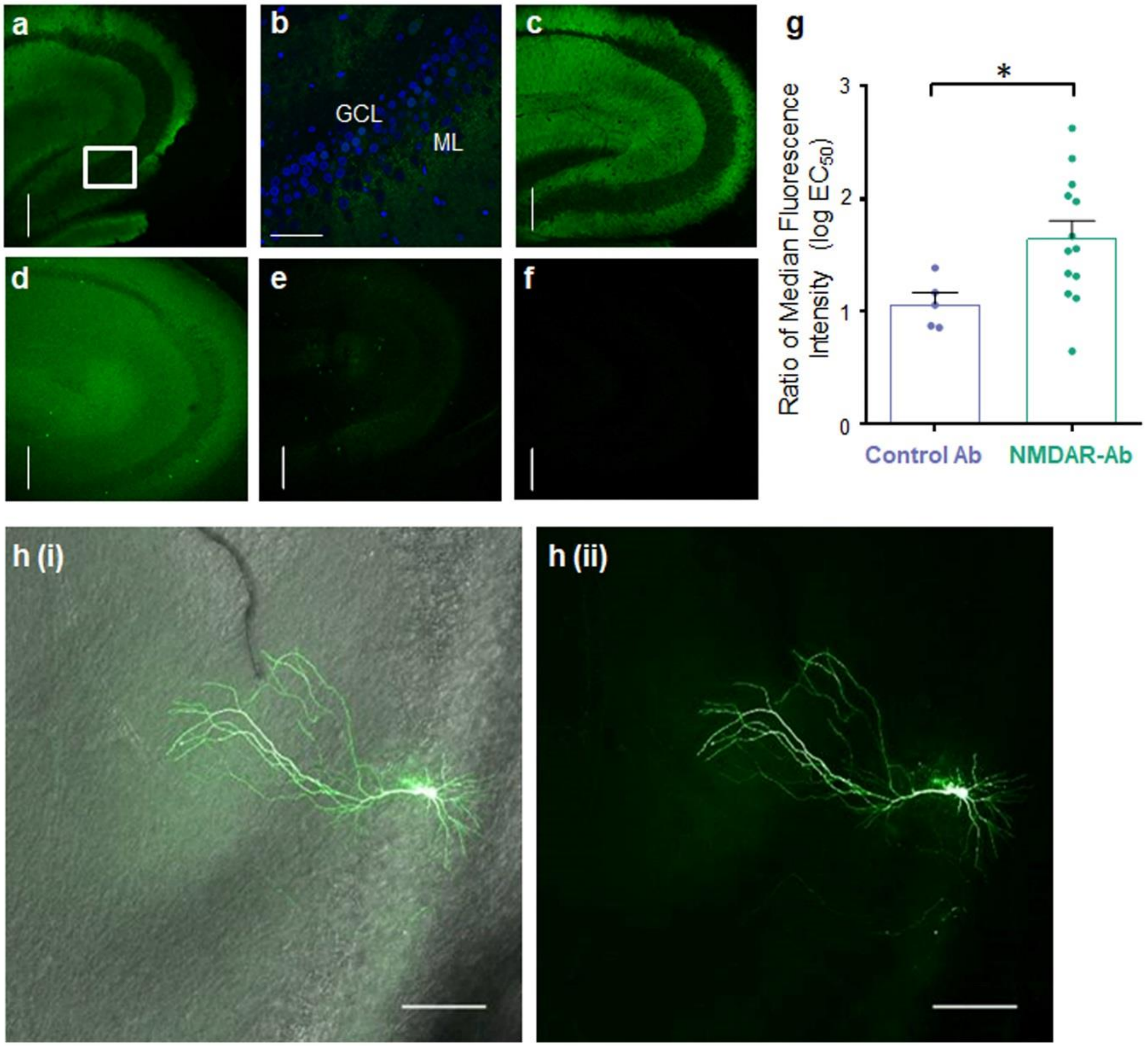

Supplementary Figure 1. Immunohistochemistry confirming NMDAR antibody binding to juvenile Wistar rat hippocampus and morphology of pyramidal cells in CA3 used for whole-cell patch clamp recordings.

a Representative confocal image of hippocampus from sagittal brain slice prepared after acute ICV injection of monoclonal NMDAR antibody SSM5 shows typical staining pattern with secondary anti-human IgG (green). Scale bar = 250µm. **b** Magnification of panel (**a**) shows the typical binding pattern of NMDAR antibodies with relative sparing of the granular cell layer (GCL) compared to the molecular cell layer (ML). Scale bar = 50µm. **c** Representative confocal image of hippocampus from sagittal brain slices prepared after chronic infusion of monoclonal NMDAR antibody 12D7 after application of secondary anti-human IgG (green). Scale bar = 250µm. **d** Representative confocal image of hippocampus from sagittal brain slice prepared after chronic infusion of NMDAR antibody positive IgG also shows typical staining pattern with

secondary anti-human IgG (green). Scale bar = 250µm. **e** Representative confocal image of hippocampus from sagittal brain slices prepared after chronic infusion of healthy control IgG after application of secondary anti-human IgG (green). There is no specific binding of these antibodies. Scale bar = 250µm. **f** Representative confocal image of hippocampus from sagittal brain slices prepared after acute injection control monoclonal antibody mG053 after application of secondary anti-human IgG (green). There is no specific binding of these antibodies. Scale bar = 250µm. **g** The NMDAR antibody treated brain slices showed increased median fluorescent intensity log EC_{50} ratios (GCL vs. ML) compared to controls (control group contains: healthy control IgG n=2 animals, 6 brain slices and control monoclonal 12D7 n=3 animals, 9 brain slices; NMDAR group contains: SSM5 monoclonal n=8 animals, 22 brain slices, 003-102 n=2 animals, 2 brain slices and patient IgG n=3 animals 5 brain slices; p=0.035, Mann-Whitney). **h** Cells used for spontaneous excitatory and inhibitory current recordings were from putative pyramidal cells with the CA3 region; an example cell injected with neurobiotin shows clear features of a pyramidal cell (green, anti-streptavidin IgG). Scale bar 150 µm.

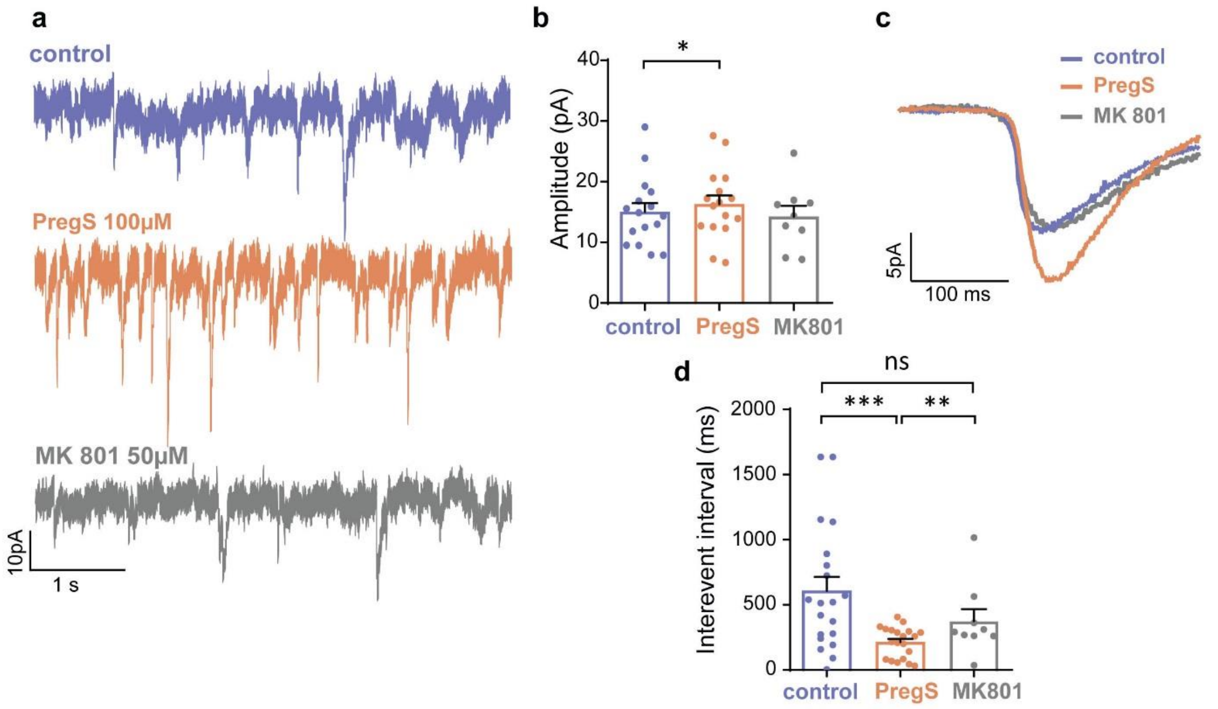

Supplementary Figure 2. Pregnenolone sulphate increases synaptic levels of NMDARs in naïve rat hippocampal neurons and glutamatergic neurotransmission in rat brain slices *in vitro*.

a Representative whole-cell patch clamp sEPSC recordings from CA3 pyramidal cells in untreated (no antibody) hippocampal slices. Scale bar 10pA vs. 1s. **b** Amplitude (pA) of sEPCS recordings from putative CA3 pyramidal neurons before and after addition of PregS (n=16 cells; p= 0.04, Wilcoxon-paired rank test); and of MK801 (n=9 cells; p=0.1, Wilcoxon-paired rank test). **c** Representative averaged amplitude (pA) of sEPSCs after addition of PregS and MK801 Scale bar 5pA vs. 100ms. **d** Interevent interval (ms) of sEPSCS recordings from putative CA3 pyramidal neurons before (control) and after addition of PregS (n=20; p=<0.001, Wilcoxon paired rank test) and of MK801 (n=9; p=0.01, Wilcoxon paired rank test).

Supplementary Table 1. Details of antibodies used in each experiment.

	Number of animals used with specified antibody					
Procedure	Healthy control IgG	Control monoclonal antibody mG053	Control monoclonal antibody 12D7	NMDAR monoclonal antibody SSM5	NMDAR monoclonal antibody 003-102	NMDAR antibody patient IgG
ICV injection	N=5/8	-	N=3/8	N=11/14	-	N=3/14
Osmotic pump infusion with EEG transmitter	N=3/6	N=3/6	-	N=2/6	N=2/6	N=2/6

Abbreviations: ICV intraventricular injection NMDAR N-methyl-D-aspartate receptor

Supplementary Table 2. Patient details for human tissue electrophysiology experiments.

Patient	Operation	Medication	Histology
1	Frontal resection	Topiramate<br>Clobazam	Focal cortical dysplasia Type IIB
2	Frontal resection	Lamotrigine<br>Carbamezapine<br>Clobazam	Focal cortical dysplasia Type IB
3	Frontal resection	Modified KD<br>Sodium valproate<br>Vigabatrin	No definite abnormal histology
4	Temporal parietal occipital disconnection	Topiramate<br>Clobazam	No definite evidence of epileptogenic lesion
5	Temporal lobectomy	Lamotrigine	Changes consistent with epilepsy
6	Temporal lobectomy	Carbamazepine<br>Levetiracetam	Hippocampal sclerosis
7	Right hemispherotomy	Carbamazepine<br>Levetiracetam<br>Clonazepam	Hippocampal sclerosis

Abbreviations: KD ketogenic diet

Supplementary References

1. Friston KJ, Bastos A, Litvak V, Stephan KE, Fries P, Moran RJ. DCM for complex-valued data: cross-spectra, coherence and phase-delays. NeuroImage. 2012;59(1):439-55.
2. Bastos AM, Usrey WM, Adams RA, Mangun GR, Fries P, Friston KJ. Canonical microcircuits for predictive coding. Neuron. 2012;76(4):695-711.
3. Penny WD. Comparing dynamic causal models using AIC, BIC and free energy. NeuroImage. 2012;59(1):319-30.

Supplementary Movies

Supp Movie 1: Hyperexcitable phenotype captured 48 hours after NMDAR antibody ICV injection in juvenile Wistar rats

Supp Movie 2: Example video clips of epileptic myoclonic jerks in NMDAR antibody chronically infused juvenile Wistar rats during wakefulness

Supp Movie 3: Example video clips of hyper excitability and epileptic myoclonic jerks in NMDAR antibody chronically infused juvenile Wistar rats during wakefulness

Supp Movie 4: Example video clips of epileptic activity during sleep in NMDAR antibody chronically infused juvenile Wistar rats
