# Supplementary material for: Multimodal electrophysiological analyses reveal that reduced synaptic excitatory neurotransmission underlies seizures in a model of NMDAR antibody-mediated encephalitis
Source: Commun Biol. 2021 Sep 20;4:1106. doi: 10.1038/s42003-021-02635-8 (PMC8452639; doi:10.1038/s42003-021-02635-8)
Supplement: Supplementary file 9 — Reporting Summary [file 42003_2021_2635_MOESM9_ESM.pdf]

nature research

manuscript number: COMMSBIO-21-0074-T
Corresponding author(s): Sukhvir Wright
Last updated by author(s): 9/8/2021

Reporting Summary

Nature Research wishes to improve the reproducibility of the work that we publish. This form provides structure for consistency and transparency in reporting. For further information on Nature Research policies, see our Editorial Policies and the Editorial Policy Checklist.

Statistics

For all statistical analyses, confirm that the following items are present in the figure legend, table legend, main text, or Methods section.

n/a	Confirmed	
☐	☒	The exact sample size (*n*) for each experimental group/condition, given as a discrete number and unit of measurement
☐	☒	A statement on whether measurements were taken from distinct samples or whether the same sample was measured repeatedly
☐	☒	The statistical test(s) used AND whether they are one- or two-sided<br>*Only common tests should be described solely by name; describe more complex techniques in the Methods section.*
☒	☐	A description of all covariates tested
☒	☐	A description of any assumptions or corrections, such as tests of normality and adjustment for multiple comparisons
☐	☒	A full description of the statistical parameters including central tendency (e.g. means) or other basic estimates (e.g. regression coefficient) AND variation (e.g. standard deviation) or associated estimates of uncertainty (e.g. confidence intervals)
☐	☒	For null hypothesis testing, the test statistic (e.g. *F*, *t*, *r*) with confidence intervals, effect sizes, degrees of freedom and *P* value noted<br>*Give P values as exact values whenever suitable.*
☐	☒	For Bayesian analysis, information on the choice of priors and Markov chain Monte Carlo settings
☒	☐	For hierarchical and complex designs, identification of the appropriate level for tests and full reporting of outcomes
☒	☐	Estimates of effect sizes (e.g. Cohen's *d*, Pearson's *r*), indicating how they were calculated

Our web collection on statistics for biologists contains articles on many of the points above.

Software and code

Policy information about availability of computer code

Data collection	Commercial: Clampex 10.4; Spike 2 8.09; LasX;<br>Opensource: Neuroarchiver software (OSI)
Data analysis	Cmmercial: Matlab R2020A; Graphpad Prism 8.0.1; Axograph X;Fiji ImageJ;Adobe Illustrator 2020<br>Opensource: Neuroarchiver software (OSI)<br>Dynamic causal modelling: SPM12

For manuscripts utilizing custom algorithms or software that are central to the research but not yet described in published literature, software must be made available to editors and reviewers. We strongly encourage code deposition in a community repository (e.g. GitHub). See the Nature Research guidelines for submitting code & software for further information.

Data

Policy information about availability of data

All manuscripts must include a data availability statement. This statement should provide the following information, where applicable:
- Accession codes, unique identifiers, or web links for publicly available datasets
- A list of figures that have associated raw data
- A description of any restrictions on data availability

Analysis code and raw data are available online at http://doi.org/ 2010.17605/OSF.IO/GUPBF and Excel sheet with source data for graphs included as Supplementary File 1

Field-specific reporting

Please select the one below that is the best fit for your research. If you are not sure, read the appropriate sections before making your selection.

- [x] Life sciences
- [ ] Behavioural & social sciences
- [ ] Ecological, evolutionary & environmental sciences

For a reference copy of the document with all sections, see nature.com/documents/nr-reporting-summary-flat.pdf

Life sciences study design

All studies must disclose on these points even when the disclosure is negative.

Sample size	In a typical in vitro electrophysiology experiment aimed at showing changes in receptor function in response to antibodies, and/or drugs, a power calculation at an alpha value of 0.8 and with P<0.05 would require 8 recorded cells/local field potential recordings in each group to achieve robust results. This was our aim for each experiment. On occasion less cells were accepted based on experimental outcomes and availability of tissue. In the forefront of our practice is to ensure that there is no unnecessary animal use. With continuous EEG recording 24 hours a day, 7 days a week, and subsequent analysis of hourly intervals of ictal events we can obtain significant quantities of data from each animal. With the chronic antibody infusion study, we have obtained robust data from 6 animals in each group for NMDAR and our other GABAAR AE models, and previous NMDAR-Ab work.
Data exclusions	No data exclusions.
Replication	The reproducibility of the data was maintained by using different NMDAR-Ab preparations.
Randomization	Randomisation was not applicable to this study design.
Blinding	Authors were blinded to the antibody infused and for data analysis.

Reporting for specific materials, systems and methods

We require information from authors about some types of materials, experimental systems and methods used in many studies. Here, indicate whether each material, system or method listed is relevant to your study. If you are not sure if a list item applies to your research, read the appropriate section before selecting a response.

Materials & experimental systems

n/a	Involved in the study	
[ ]	[x]	Antibodies
[x]	[ ]	Eukaryotic cell lines
[x]	[ ]	Palaeontology and archaeology
[ ]	[x]	Animals and other organisms
[ ]	[x]	Human research participants
[x]	[ ]	Clinical data
[x]	[ ]	Dual use research of concern

Methods

n/a	Involved in the study	
[x]	[ ]	ChIP-seq
[x]	[ ]	Flow cytometry
[x]	[ ]	MRI-based neuroimaging

Antibodies

Antibodies used	Three NMDAR-Ab preparations were used, and three control antibody preparations. All were previously used in research studies concerning NMDAR-Abs (Wright et al 2015; Kreye et al 2016; Malviya 2017), and use is detailed in Supplementary Table 1 in the manuscript.
Validation	The validation for use of these antibodies is provided within the manuscript. All antibodies had been previously used in studies specified and known to be pathogenic.

Animals and other organisms

Policy information about studies involving animals; ARRIVE guidelines recommended for reporting animal research

Laboratory animals	Wistar rats, male, post natal age 21 days
Wild animals	The study did not involve wild animals
Field-collected samples	The study did not use field collected samples
Ethics oversight	All animal experiments were conducted in compliance with UK Home Office guidelines and ARRIVE guidelines.

Note that full information on the approval of the study protocol must also be provided in the manuscript.

Human research participants

Policy information about studies involving human research participants

Population characteristics	Human tissue samples were used for one experiment. The patients were recruited from Birmingham Children's Hospital. 7 patients' tissue samples were used, 4 females, 3 males, median age was 6 years (range 4-16 years). Further relevant clinical data is provided in Supplementary Table 2.
Recruitment	Patients were recruited through the Childrens Epilepsy Surgery Service programme.
Ethics oversight	The study protocol was approved by the Black Country Local Ethics Committee, Aston University Ethics Committee and Birmingham Children's Hospital R&D Dept. Full information is provided in the manuscript

Note that full information on the approval of the study protocol must also be provided in the manuscript.
